# Analysis of *Elymus nutans* seed coat development elucidates the genetic basis of metabolome and transcriptome underlying seed coat permeability characteristics

**DOI:** 10.3389/fpls.2022.970957

**Published:** 2022-08-18

**Authors:** Jing Zhou, Yan Li, Xun Wang, Yijia Liu, Rakefet David-Schwartz, Mira Weissberg, Shuiling Qiu, Zhenfei Guo, Fulin Yang

**Affiliations:** ^1^National Engineering Research Center of Juncao Technology, Fujian Agriculture and Forestry University, Fuzhou, China; ^2^College of Animal Sciences (College of Bee Science), Fujian Agriculture and Forestry University, Fuzhou, China; ^3^Qinghai University, Academy of Animal Science and Veterinary Medicine, Xining, China; ^4^Volcani Center, Agriculture Research Organization, Institute of Plant Sciences, Beit Dagan, Israel; ^5^College of Agro-Grassland Science, Nanjing Agricultural University, Nanjing, China

**Keywords:** seed coat, permeability, development, metabolome, transcriptome, *Elymus nutans*

## Abstract

The seed coat takes an important function in the life cycle of plants, especially seed growth and development. It promotes the accumulation of nutrients inside the seed and protects the seed embryo from mechanical damage. Seed coat permeability is an important characteristic of seeds, which not only affects seed germination, but also hinders the detection of seed vigor by electrical conductivity (EC) method. This research aimed to elucidate the mechanism of seed coat permeability formation through metabolome and transcriptome analysis of *Elymus nutans*. We collected the samples at 8, 18, and 28 days post-anthesis (dpa), and conducted a seed inclusion exosmosis experiment and observed the seed coat permeability. Moreover, we analyzed the changes in the metabolome and transcriptome during different development stages. Here, taking 8 dpa as control, 252 upregulated and 157 downregulated differentially expressed metabolites (DEMs) were observed and 886 upregulated unigenes and 1170 downregulated unigenes were identified at 18 dpa, while 4907 upregulated unigenes and 8561 downregulated unigenes were identified at 28 dpa. Meanwhile, we observed the components of ABC transporters, the biosynthesis of unsaturated fatty acids, and phenylalanine metabolism pathways. The key metabolites and genes affecting seed coat permeability were thiamine and salicylic acid. Furthermore, there were 13 and 14 genes with correlation coefficients greater than 0.8 with two key metabolites, respectively, and the —log_2_Fold Change— of these genes were greater than 1 at different development stages. Meanwhile, pathogenesis-related protein 1 and phenylalanine ammonia-lyase play an important role in regulating the formation of compounds. Our results outline a framework for understanding the development changes during seed growth of *E. nutans* and provide insights into the traits of seed coat permeability and supply a great significance value to seed production and quality evaluation.

## Introduction

As part of the seeds, the seed coat or testa, takes an important function in the growth and development of plants (Ben-Tov et al., 2015). It can protect the embryo from mechanical damage and pathogen infection in mature seeds and store nutrients to prevent loss in developing seeds (Mendu et al., 2011; Ezquer et al., 2016). The capacity of the testa to defend embryo tissue away from mechanical and oxidative stress is important for seed viability (Chen et al., 2014b; Loubéry et al., 2018). The seed coat may be used in an aqueous solution as a immerse or during priming to inject active substance into the embryo (Salanenka and Taylor, 2011). In seed coats, permeability is a common phenomenon. The permeability of the seed coat is generally considered to control the exchange of water, gas, and nutrients between the seed embryo and the outside world and plays a prominent part in seed germination, dormancy, storage, and species reproduction (Dongen et al., 2001; Briggs and Morris, 2008; Li et al., 2010). Different plant seed coats have different osmotic characteristics. According to the osmotic capacity of different substances, seed coat permeability is generally divided into three types: non-permeable, semi-permeable, and permeable. Non-permeable seed coats are one of the main factors causing seed dormancy (physical dormancy), which is mainly because the tight seed coat structure makes it difficult for the seed to absorb water and air (Jayasuriya et al., 2007; Orozco-Segovia et al., 2007; Brancalion et al., 2010). Semi-permeable seed coats ordinarily have selective permeability to the exchange of materials inside and outside the seed coat. They can absorb water and swap gas, but they can limit the permeation of macromolecules (Beresniewicz et al., 1995; Amritphale et al., 2010; Zhou et al., 2013b). Permeable seed coats allow not only the exchange of water and gas, but also the infiltration of some large numbers of macromolecular substances (Barua et al., 2012).

Seed coat permeability is affected by the environment of seed development, such as maternal temperature signaling, which regulates seed coat permeability properties. In soybeans, high temperatures during grain filling raise the occurrence of impermeable seed coat (hard seed), which is related to disunion and deferred emergence and germination (Kebede et al., 2014). On the contrary, in *Arabidopsis thaliana*, lower maturation temperatures augments testa permeability to tetrazolium, and mutants with superior seed coat permeability cannot easily enter strongly dormant states (MacGregor et al., 2015). Meanwhile, low-temperature plasma treatment modifies *A. thaliana* testa permeability due to the change of lipid compounds and structure (Bafoil et al., 2019). In addition to temperature, water content is another main factor influence on permeability. The testa permeability of *Astragalus adsurgens* was induced by reduced moisture content, which caused seed dormancy (Jaganathan et al., 2019). The seed coat permeability is also determined by testa thickness and composition. The cell wall thickness in the outer integuments varies according to seed development. Along with a build-up of polyphenolic compounds, callose, and lipids cause changes in seed coat permeability (Janská et al., 2019).

Seed coat permeability was generally thought to contribute toward seed treatment, especially in seed detection. Under eco-friendly pest management, the penetration ability of tomato seed coat is the key factor for exogenous jasmonic acid to successfully mediate the pest defense system (Mouden et al., 2020). Different permeability characteristics make it difficult to measure seed vigor using the electrical conductivity (EC) and tetrazolium chloride (TTC) staining methods. In the detection of orchid seed vigor, the TTC method cannot accurately identify whether the seed is actually alive; thus, a rapid method to evaluate the permeability level of seed coat needs to be developed (Magrini et al., 2019). In previous studies, the TTC and EC methods were used to determine the seed vigor of rice seeds with whole testa and poaceae seeds with punctured seed coat. The results demonstrated that the seed coat permeability was the primary factor limiting the application of seed vigor detection method (Sun et al., 2015, 2018).

Many studies on seed coat permeability have been conducted. However, most of them focus on understanding its characteristics, and few have investigated the regulation mechanism of seed coat permeability. In *Arabidopsis*, the content of condensed-tannin decreased seed permeability to tetrazolium dye, while *rgl2-1* mutants showed increased seed coat permeability; suberin acted as a hydrophobic obstacle to control the movement of gases, water, and solutes and as an antimicrobial obstruction (Vishwanath et al., 2013; Chen et al., 2014a). Transparent testa (*tt*) mutations of *Arabidopsis* have anomalously high seed coat permeability because the cuticles of developing *tt* mutant integument have meaningful structural faultiness, which are related to increasing cuticle permeability (Loubéry et al., 2018). Phenylpropanoid gene expression in seeds was correlated with high concentrations of seed coat procyanidins, which leads to decreased seed coat permeability (MacGregor et al., 2015). Molecular mapping and inheritance studies identified a single dominant gene *isc* (impermeable seed coat) that may be important in developing cultivars of soybean with permeable seed coat (Kebede et al., 2014). Seed coat impermeability is controlled by the product of genes (*GmHs1-1*, *qHS1*) that encode calcineurin-like transmembrane proteins, endo-1,4-β-glucanase and β-1,4-glucans, which reinforce testa impermeability (Jang et al., 2015; Sun et al., 2015; Janská et al., 2019).

*Elymus nutans* is a kind of perennial gramineous forage, which is widely distributed in the alpine region at an altitude of 3,000–5,000 m (Fu et al., 2014; Liu et al., 2019). It has strong cold resistance and can safely overwinter at low temperature (Ding et al., 2020). *E. nutans* has high yield, long growing season, and drought and trample resistance, which makes it an important grass species for grassland establishment and ecological environment construction in the alpine region (Fu et al., 2014). Previous studies have found that *E. nutans* seed coat is semi-permeable, making it difficult for EC and TTC methods to detect seed vigor. The semi-permeable property of the seed coat is owing to the existence of a special structure called the semi-permeable layer. This layer can restrict or impede the exchange of solutes while permitting the penetration of external and internal water and gas, which provides valuable protection to support plant health and protect plant growth, development, and germination.

However, in *E. nutans*, the semi-permeable layer is situated in the outermost layer of the seed coat and originates from the outer layer of the inner integument at 10 days post-anthesis (dpa) (Zhou et al., 2013b). The purpose of this study is to reveal the main substances and key genes associated with *E. nutans* seed coat permeability variation using metabolome and transcriptome methods and to provide a theoretical basis for the formation mechanism of this characteristic. The results will supply a great significance value to seed production and quality evaluation.

## Materials and methods

### Experimental materials

#### Seed collection

The seeds of wild *E. nutans* were taken from a field place: Zeku Country, Qinghai Province, China (100°58′E, 35°23′N), from July to August 2020. The region belongs to a typical semi-temperate climate and the general characteristics are low temperature, insufficient heat, short frostless period and strong solar radiation. Totally 1,000 spikelets with flowers were tagged at anthesis, and samples were collected at 8, 18, and 28 dpa. The seeds at each development stage were divided into three parts. One part was dried naturally to test its physiological indicators, including lanthanum trace analysis. In the second part, the lemmas were removed from fresh seeds, and the seeds were fixed in 4% glutaraldehyde for observation of seed coat structure. In the third part, the seed coat was removed from the naked seed with a sharp scalpel and immediately fixed in liquid nitrogen for metabolome and transcriptome analysis.

#### Seed quality index

Thousand-seed weight was measured from the average weight of eight repetitions of 100 seeds. The petri dish paper germination method was used to test seed germination percentage on four repetitions of 50 seeds in each development stage incubated in a light incubator (GXZ-280B, Ningbo, China) at 25°C according to GB/T 2930.4-2017. The number of germinating seeds was counted every day during the experiment, and the seedlings were kept in the petri dish. The seed germination rate was calculated at the end of 12 days. On the basis of the above germination test, the germination energy, vigor index, and seedling and root length of seedlings grown in the first 5 days were calculated. The germination energy is the cumulative number of germinations in the first 5 days divided by the sum of tested seeds multiplied by 100%. The vigor index is the sum of the average seedling length and average root length multiplied by the germination percentage. The seed moisture content was determined using the high-constant-temperature oven method described by the International Seed Testing Association.

### Seed coat permeability characteristic

#### Electrical conductivity and imbibition rate testing

The EC and imbibition rate were determined using four repetitions of 50 seeds from three development stages. According to the Hampton and TeKrony method, each replicate was weighed, shifted to 100 mL distilled water, and stored at 20°C for 24 h. A conductivity meter (DDSJ-319L, Shanghai, China) was used to test conductivity. Then, the seeds were surface desiccated and weighed. The imbibition rate was indicated to the wet weight minus the dry weight divided by the dry weight.

#### Light microscopy and lanthanum tracer energy dispersive X-ray analyses

The fixed caryopsis coat including endosperm tissue was embedded in Technovit7100 and cut into 1 μm semi-thin slices according to the methods described by Zhou et al. (2013a). The slices were stained with 0.05% aniline blue in 0.1 M phosphate buffer (pH 8.2) for 20 min. The structure of the caryopsis coat was examined, and images were taken by means of a compound microscope (Eclipse E 100, Nikon, Tokyo, Japan).

In order to detect the penetration characteristics of the seed coat, lanthanum nitrate tracer method combined with energy dispersive X-ray (EDX) technology was used to observe the permeability of lanthanum in seeds. The dried seeds without lemmas were soaked in distilled water for 20 h at 20°C, and the seeds with intact seed coat were selected using a stereomicroscope (Zoom-600, Shanghai, China). The intact seeds were incubated in 1% (W/V) lanthanum nitrate solution at 20°C. After 24 h, the seeds were taken out and dried naturally. The dried seeds were cut into halves with a razor blade and fixed to an aluminum shell. The structure of the seeds was observed using the JEM 5600 LV scanning electron microscope (SEM) with EDX spectroscopy (KEVE2, United States). Then, the seeds were sputter coated and viewed under the backscattered electron imaging mode at 20 kV. The snowflake crystals located in seed samples were analyzed for 20 s. Lanthanum nitrate amounts were based on the area of L-series.

#### Fluorescence staining indicated seed coat permeability

To further examine the seed coat permeability, the dry seeds without lemmas were immersed in 20°C distilled water for 24 h. Then, the seeds of three development stages with intact seed coat were placed in 1% Rhodamine B prepared with absolute alcohol for 24 h. Thereafter, the penetration of the fluorescent agent into the seeds was examined using a fluorescence microscope (Leica MDG41, Singapore).

### Non-targeted metabolite analysis of seed coat permeability

#### Metabolite extraction

About 50 mg of seed coat of *E. nutans* was placed in a 1.5 mL eppendorf tube for non-targeted metabolite profiling with four replicates per development stage. All samples were added with 800 μL of methanol and ground for 90 s by using a high-throughput tissue grinding mill (SCIENTZ-48, Zhejiang, China). The mixture was blended by ultrasound for 30 min and centrifuged at 4°C for 15 min at 12,000 rpm. Then, 5 μL DL-o-Chlorophenylalanine was added to the supernatant and transferred into the sample vials.

#### Non-targeted metabolite analysis by LC-MS/MS

Metabolites were separated using an ultra-high-performance liquid phase chromatograph (UHPLC, Waters ACQUITY Ultra Performance LC, United States) equipped with the ACQUITY UPLC HSS T3 Column (100 mm× 2.1 mm × 1.8 μm). The mobile phase A was 0.05% formic acid, and the mobile phase B was acetonitrile. The mobile phase was used to separate the metabolites while the flow rate was maintained at 300 μL/min. The parameters of gradient elution were set as follows: 0 min, 95% A, 5% B; 1 min, 95% A, 5% B; 12 min, 5% A, 95% B; 13.50 min, 5% A, 95% B; 13.60 min, 95% A, 5% B; 16 min, 95% A, 5% B. The injection volume was 4 μL and the automatic temperature was 4°C.

Then, the analytes were detected by using a Q Exactive™ Mass Spectrometer (MS/MS, Thermo Fisher Scientific, United States) with electron spray ionization including positive and negative ion mode to obtain mass spectral data, which were collected by full scan mode with m/z 70–1050 and dd-MS2 mode with TopN = 10, respectively. The following settings were used: sheath gas flow rate, 45 Arb; auxiliary gas flow rate, 15 Arb; capillary temperature, 350°C; full ms resolution, 70,000; MS/MS resolution, 17,500; collision energy, 15/30/45 eV; spray voltage, 3.0 kV (positive) or –3.2 kV (negative). In order to enhance the accuracy of identified substance, the following criteria were used: accurate mass with variation less than 10 ppm and MS/MS spectra with high forward and reverse scores based on comparisons of the ions present in the experimental and library spectrum entries (Shen et al., 2020). The metabolites were identified with mzCloud, FiehnLib and Chinese Natural Product databases.

#### Data preprocessing and annotation

Preprocessing and quality assessment of raw data were performed. Single peaks and total ion current of each sample were filtered and normalized with R package metaX. The missing value in the original data was simulated by half of the minimum value. Then, the low mass ions were removed, and ions with relative standard deviation >30% were filtered out. After obtaining the matrix data, the original data were annotated into the KEGG,^1^ HMDB,^2^ METLIN,^3^ ChEBI,^4^ and PubChem^5^ databases.

The resulting three-dimensional data involving the sample name, peak number, and normalized peak area were supplied to R package metaX for principal component analysis (PCA) and orthogonal partial least square-discriminant analysis (OPLS-DA). PCA exhibited the distribution of original data. To acquire a higher level of group separation and a better understanding of the variables responsible for classification, supervised OPLS-DA was applied. The first principal component of variable importance in projection (VIP) was obtained.

#### Identification of differential metabolites and enrichment analysis

We used a combination of the *p*-values of Student’s *t*-tests with the VIP value to filter DEMs, with extra screening criteria (*p* < 0.05, VIP > 1). In addition, commercial databases including KEGG were used to search for the pathways of DEMs, which were identified by the second mass spectrometry.

### Characteristics of transcriptome in seed coat permeability

#### Total ribonucleic acid extraction and sequencing library preparation for transcriptome

High-quality total ribonucleic acid (RNA) was extracted from the *E. nutans* seed coat using the TRIzol reagent (TransGen, Beijing, China) following the manufacturer’s instructions. The purity and quality of RNA were gauged and checked. The RIN value of every sample was greater than 8.4. Every development stage had four replicates. About 1.5 μg of RNA was used to build the RNA-seq library with Illumina’s NEBNext^®^ Ultra™ RNA Library Prep Kit (Illumina Inc., San Diego, CA, United States). The purified mRNA was carried out by divalent cations. First and second-strands cDNAs were synthesized using random hexamer primer and DNA Polymerase I and RNase H (Invitrogen, Carlsbad, CA, United States), respectively. The remaining molecules were transformed into blunt ends via exonuclease/polymerase activities. The library preparations were sequenced on an Illumina HiSeq 4000 platform and generated paired-end reads of 150 bp.

#### Sequence read mapping and *de novo* assembly

Raw data in FASTQ format were first processed to filter out rRNA by using bowtie2 (Langmead and Salzberg, 2013). Clean data were acquired by removing reads containing poly-N, adapter, and low-quality reads from raw data through in-house Perl scripts. At the same time, GC-content, Q20, Q30, and sequence duplication level of clean data were calculated. According to the Trinity method including the Inchworm, Chrysalis and Butterfly program, the spliced transcript was used as the reference sequence. Clean reads were separately mapped to them using SALMON software (version 0.14.1)^6^ and *de novo* assembled with parameters of Kmer = 25 (Grabherr et al., 2011). Then, CD-HIT,^7^ Corset and BUSCO software were used to classify transcripts, remove redundancy, and evaluate the quality of splicing, respectively.

#### Gene functional annotation, differential expression analysis, and transcription factor identify

All the assembled transcripts were analyzed by BLAST with seven public databases, including non-redundant protein sequence (Nr), nucleotide database (Nt), Pfam protein families database (Pfam), manually annotated and reviewed protein sequence database (Swiss-Prot), eukaryotic orthologous groups database (KOG), Gene Ontology database (GO), and Kyoto Encyclopedia of Genes and Genomes pathway database (KEGG). The expression levels of unigenes were standardized and calculated as the values of fragments per kilobase of transcripts per million mapped fragments (FPKM) during the assembly and clustering process (Trapnell et al., 2010). After normalizing the read count data with DESeq (Anders and Huber, 2010), the *p*-values of DESeq2 package analyses were adjusted via the Benjamini–Hochberg method to determine the false discovery rate (FDR) and identify differentially expressed genes (DEGs). Then, the *p*-value obtained from the test was corrected to obtain the *q*-value. The standard of differential gene expression screening is *q*-value < 0.05 (Robinson et al., 2010). To study the seed development significance of DEGs, the GO database was used for GO enrichment analysis of DEGs during the different processing of *E. nutans* using the GOseq (v1.22) software (Young et al., 2010). By estimating the bias of gene length, the probability of GO term enriched by different genes can be calculated more accurately. The assorted metabolic pathways of DEGs were analyzed by using the KEGG database (Mao et al., 2005). The statistical enrichment of DEGs was tested using KOBAS 2.0 web server, and a corrected *p*-value < 0.05 was considered to be significantly enriched in KEGG. All identified DEGs were blasted with PlantTFDB 4.0.^8^ We chose prediction interface to input unigenes sequence in fasta format. Both the hmmscan and blast *e*-value were set as 1 × e^–5^.

#### Time series analysis in seed coat development

To explore the new important unigenes that can affect seed coat penetration, time series analysis was conducted to excavate the transcription data. We removed genes whose FPKM values were less than 1 in all samples. Then, we used the log normalize data strategy to transform the data and selected STEM (v1.3.13) clustering for time series analysis. Subsequently, we drew the time sequence diagram. The maximum number of modules is 20 (the default is 50), the maximum change multiple is 2, and other parameters are default.

#### Quantitative RT-PCR validation

Quantitative RT-PCR analysis was processed on a CFX Connect qPCR detection system to verify the accuracy of the RNA-seq results. Six genes were randomly detected, and the *EnACT* gene was used as a house-keeping gene (the primers are shown in Supplementary Table 1; Niu et al., 2017). The qPCR program run consisted of an initial denaturation step at 95^°^C for 15 min followed by amplification and quantification cycles repeated 40 times at 95^°^C for 10 s, 58^°^C for 20 s and 72^°^C for 30 s. We used the 2^–Δ^ ^Δ^ *^CT^* method to calculate the relative expression levels of genes.

### Co-expression analysis of metabolome and transcriptome

The obtained DEMs and DEGs data were applied in the calculation of correlation coefficients (Spearman rank correlation test) using R through inhouse Perl script. Positive and negative correlations >0.8 and <–0.8 were considered for the construction of a dynamic network. The results were visualized using Cytoscape (version 3.7.1) and Origin 2018.

### Statistical analysis

Statistical analyses of physiological data were performed using SPSS 20.0 (SPSS Inc., Chicago, IL, United States). The significance of differences in each processing was tested by one-way ANOVA and Duncan’s multiple comparative analysis (*p* < 0.05).

## Results

### Physiological indexes and permeability characteristic during seed development of *Elymus nutans*

**FIGURE 1:**
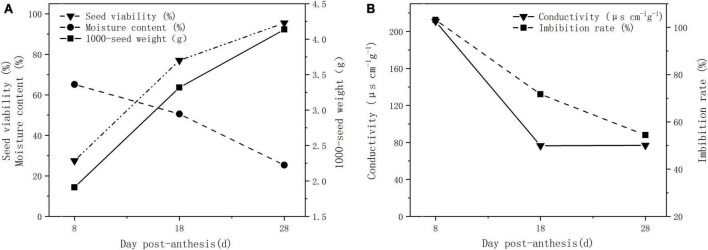
Physiological indexes of plant seeds at different developmental stages. **(A)** Seed viability, moisture content, and 1,000-seed weight. **(B)** EC and imbibition rate.

The seed viability and 1000-seed weight showed an upward trend with seed development, while moisture content, imbibition rate, and EC showed a downward trend (Figure 1). From 8 to 28 dpa, the seed viability increased from 27.50 to 95.50%, while the seed weight increased from 1.91 to 4.14 g. The seed moisture content decreased from 65.19% at 8 dpa to 25.33% at 28 dpa (Figure 1A), and the imbibition rate and EC decreased from 103.15 to 54.42% and 210.90–76.85 μs⋅ cm^–1^⋅g^–1^, respectively (Figure 1B). A pericarp, two layers of inner integument, primary aleurone cell layer and free endosperm cells were observed in the seed coat at 8 dpa (Supplementary Figure 1A). Several parenchymal cells were disappeared, and the inner integument was developed to form the compact seed coat located outside of the aleurone layer at 18 dpa (Supplementary Figure 1B). Upon reaching maturation, the seeds contained a pericarp, seed coat, aleurone layer, and endosperm. The pericarp became a membranous tissue without cellular structure, and the seed coat still adhered tightly to the aleurone cell layer at 28 dpa (Supplementary Figure 1C).

**FIGURE 2:**
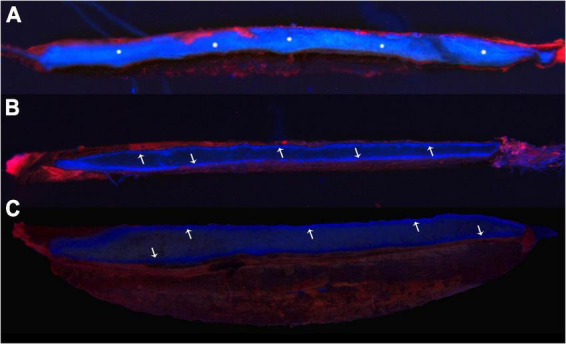
Fluorescence staining indicates the osmotic properties of the seed coat. **(A–C)** Samples at 8, 18, and 28 dpa, respectively. The snowflakes showed that the fluorescent dye infiltrated into the inside seed, and the arrows indicated that the dye was blocked outside the seed, forming a fluorescent line.

The structure of the seed coat at 18 dpa was observed using SEM (Supplementary Figure 2A). Parts a, b, and c are the outside of the seed coat, and parts d and e are the inside contents of the seed. Combined with EDX, the lanthanum peaks detected on the L-series in parts a, b, and c showed that the lanthanum was deposited on the outside of the seed (Supplementary Figures 2B–D). On the contrary, the lanthanum peak was not detected in the seed interior (Supplementary Figures 2E,F). Seed coat permeability was detected based on penetration of Rhodamine B into seeds during seed development. Fluorescence in the seeds could be observed at 8 dpa, indicating that the dye was penetrated into the inner seed (Figure 2A), but the fluorescent dye was blocked on the outside of the seed which formed a circle at 18 and 28 dpa (Figures 2B,C), indicating that the seed coat permeability decreased.

### Metabolomic changes and key metabolites associated with seed coat permeability during seed development

**FIGURE 3:**
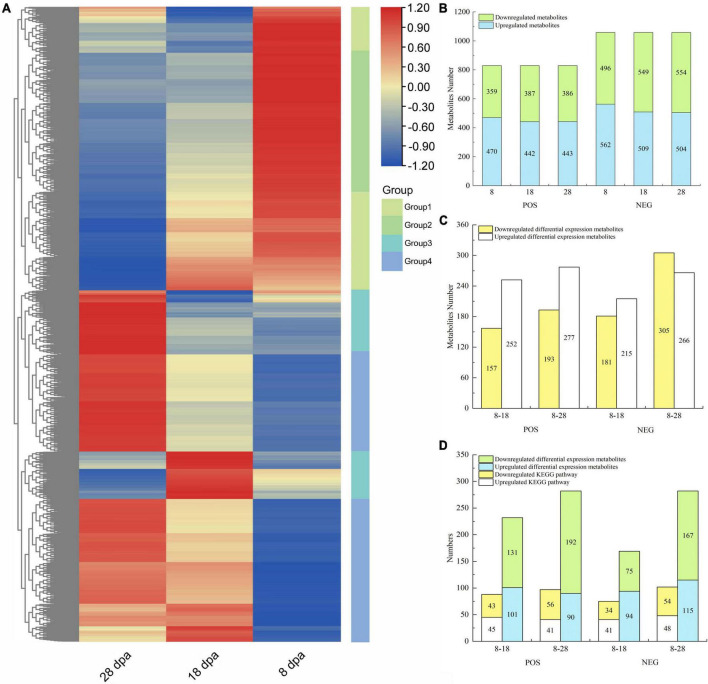
Metabolite abundance differences between three different development stages of *E. nutans* studied on the basis of LC-MS analysis. **(A)** Hierarchical clustering heatmap of all metabolites under the POS and NEG. Each column corresponded to 8, 18, and 28 dpa, and four groups were separated; **(B)** up- and downregulated metabolites under the POS and NEG; **(C)** up- and downregulated DEMs under the POS and NEG; **(D)** all DEMs enriched in KEGG and the KEGG numbers.

To characterize the metabolites associated with responsible to seed coat development in *E. nutans*, the varieties of metabolites were detected by UHPLC-MS/MS. MS signals from positive and negative ionization modes were analyzed for each sample to exclude commonly known adduct and fragment ions and to reduce signal redundancy. A total of 1887 metabolites were identified in the positive ion mode (POS) and negative ion mode (NEG) (Supplementary File 1). All the identified metabolites were classified into four groups by the hierarchical clustering heatmap (Figure 3A). There were 470, 442, and 443 upregulated and 359, 387, and 386 downregulated metabolites observed under the POS testing at 8, 18, and 28 dpa, respectively (Figure 3B). Taking 8 dpa as control, 252, 277 upregulated and 157, 193 downregulated DEMs were observed under the POS testing, respectively (Figure 3C).

KEGG analysis was performed on the DEMs identified by secondary mass spectrometry (Figure 3D). Under the POS, only 101 and 90 upregulated DEMs were annotated into 45 and 41 upregulated KEGG pathways (Figure 3D). However, under the NEG, 75 and 167 downregulated DEMs were annotated into 34 and 54 downregulated KEGG pathways (Figure 3D).

**FIGURE 4:**
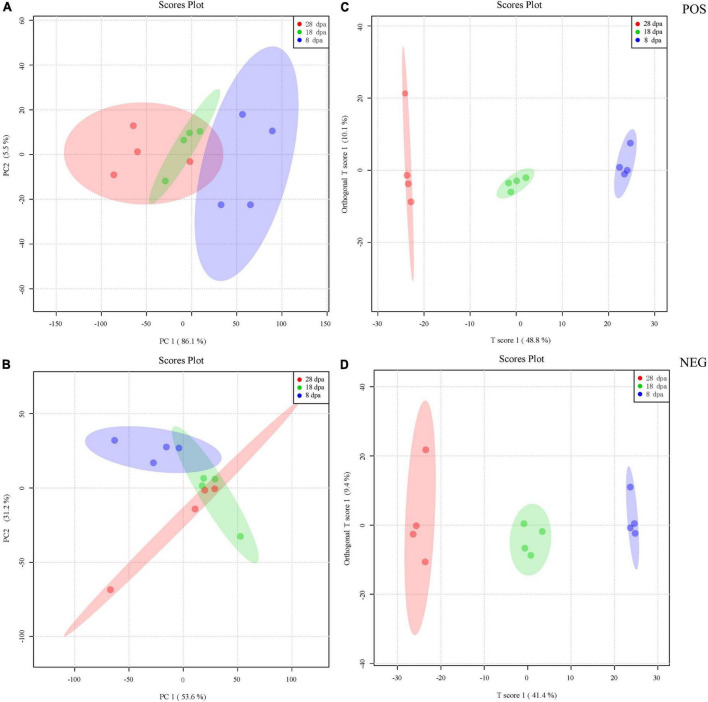
PCA and OPLS-DA score plot derived from the LC-MS spectra of extracts obtained from seed development stages at 8 (blue), 18 (green), and 28 dpa (red). **(A,B)** PCA was first used to obtain an overview of metabolic profiles of the 8, 18, and 28 dpa conditions under the POS and NEG. The different plots represent combinations of the first and second principal components (PC1 and PC2). The proportions of explained variance are shown in brackets. **(C,D)** The OPLS-DA score plot was then employed to maximize the separation according to the classification of samples at different stages of seed development acquired under the POS and NEG. The proportions of explained variance are shown in brackets. Each development stage contains four replicates.

PCA was performed on the basis of peak areas. The first principal component (PC1), which accounts for 86.1 and 53.6% of the variance separates the different development stages, while PC2 (5.5 and 31.2%) effectively separates the differential samples under the POS and NEG (Figures 4A,B). For OPLS-DA, the predicted principal component scores of the first principal component were 48.8 and 41.4%, while the orthogonal principal component scores were 10.1 and 9.4% under the POS and NEG testing, respectively (Figures 4C,D).

To accurately identify the metabolites affecting seed coat permeability, we further analyzed the substances identified by secondary mass spectrometry. According to the *p*-value < 0.05 of the KEGG annotation, the ABC transporters; biosynthesis of unsaturated fatty acids (UFA); and sulfur relay system pathways were significantly enriched under the POS analysis (Table 1). Meanwhile, under the NEG analysis, citrate cycle (TCA cycle); alanine, aspartate and glutamate metabolism; C5-branched dibasic acid metabolism; lysine degradation; and glyoxylate and dicarboxylate metabolism were the significantly enriched pathways at 8 and 18 dpa (Table 2).

**TABLE 1:** Positive analysis significant enrichment of KEGG pathway.

	POS up				POS down			
	Pathway	Hits	Raw_p	FDR	Pathway	Hits	Raw_p	FDR
8–18	ABC transporters	5	0.0006851	0.01781	Aminoacyl-tRNA biosynthesis	4	0.000117	0.00351
					Tropane, piperidine and pyridine alkaloid biosynthesis	4	0.0003347	0.003565
					Biosynthesis of amino acids	5	0.0003565	0.003565
					Lysine degradation	3	0.002006	0.01504
					2-Oxocarboxylic acid metabolism	4	0.004287	0.02322
					ABC transporters	4	0.004644	0.02322
					Valine, leucine and isoleucine biosynthesis	2	0.006163	0.02641
					Lysine biosynthesis	2	0.01397	0.05241
					Valine, leucine and isoleucine degradation	2	0.0198	0.06599
					Cyanoamino acid metabolism	2	0.02256	0.06767
					Nicotinate and nicotinamide metabolism	2	0.03282	0.08952
					Flavonoid biosynthesis	2	0.03156	0.1326
8–28	Biosynthesis of unsaturated fatty acids	2	0.04329	0.2215	Aminoacyl-tRNA biosynthesis	4	0.0002518	0.007554
	Sulfur relay system	1	0.04923	0.2215	Tropane, piperidine and pyridine alkaloid biosynthesis	4	0.0007107	0.009118
					Biosynthesis of amino acids	5	0.0009118	0.009118
					ABC transporters	5	0.001243	0.009321
					Lysine degradation	3	0.003465	0.02079
					2-Oxocarboxylic acid metabolism	4	0.008608	0.03793
					Valine, leucine and isoleucine biosynthesis	2	0.008849	0.03793
					Pantothenate and CoA biosynthesis	2	0.01482	0.05558
					beta-Alanine metabolism	2	0.01677	0.05592
					Lysine biosynthesis	2	0.0199	0.0597
					Valine, leucine and isoleucine degradation	2	0.02805	0.07651
					Cyanoamino acid metabolism	2	0.0319	0.07974
					Glycine, serine and threonine metabolism	2	0.03874	0.0894
					Nicotinate and nicotinamide metabolism	2	0.0461	0.09878

**TABLE 2:** Negative analysis significant enrichment of KEGG pathway.

	NEG up				NEG down			
	Pathway	Hits	Raw_p	FDR	Pathway	Hits	Raw_p	FDR
8–18	Phenylalanine metabolism	2	0.01433	0.09876	Citrate cycle (TCA cycle)	2	0.004671	0.09251
	Ubiquinone and other terpenoid-quinone biosynthesis	2	0.01975	0.09876	Alanine, aspartate and glutamate metabolism	2	0.009069	0.09251
	Plant hormone signal transduction	1	0.03739	0.1246	C5-Branched dibasic acid metabolism	2	0.01322	0.09251
					Lysine degradation	2	0.02749	0.1443
					Glyoxylate and dicarboxylate metabolism	2	0.04092	0.1719
8–28	Phenylalanine metabolism	2	0.02522	0.2726	Citrate cycle (TCA cycle)	2	0.005316	0.09995
	Plant hormone signal transduction	1	0.04957	0.2726	Alanine, aspartate and glutamate metabolism	2	0.0103	0.09995
					C5-Branched dibasic acid metabolism	2	0.01499	0.09995
					Lysine degradation	2	0.03107	0.1553
					Glyoxylate and dicarboxylate metabolism	2	0.04612	0.1845

**FIGURE 5:**
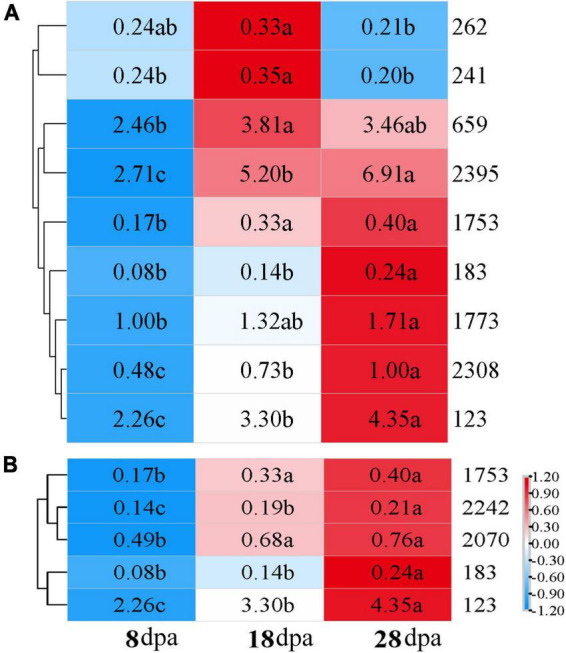
Expression of key metabolites in different samples. Distribution of key identified metabolites across different samples compared with the different development stages. Values represented in the scale correspond to the relative quantitative value of metabolites. Letter indicate the significant difference of relative quantitative values of three stages. **(A)** 8 dpa with 18 dpa; **(B)** 8 dpa compared with 28 dpa. 262: choline, 241: 4-coumaric acid, 659: L-glutamic acid, 2,395: DL-alpha-tocopherol, 1,753: thiamine, 183: hydrocinnamic acid, 1,773: adenosine, 2,308: alpha-lactose, 123: salicylic acid (SA), 2,242: docosapentaenoic acid, 2,070: eicosapentaenoic acid.

To determine the key metabolites that affected seed coat permeability, we focused on the upregulated metabolites that were significantly enriched in KEGG, and 11 metabolites exhibited different development stages under the POS and NEG (Supplementary Table 2). We performed heat-map analysis on the differential metabolites in each treatment combination and screened out compounds with more significant expression at 18 and 28 dpa than 8 dpa. Taking 8 dpa as control, 4-coumaric acid (241), L-glutamic acid (659), DL-alpha-tocopherol (2395), thiamine (1753), alpha-lactose (2308), and salicylic acid (SA) (123) were visualized in a plot, and their contents showed an upward trend at 18 dpa (Figure 5A). With seed development, thiamine (1753) and SA (123) continued to be deposited in the seed coat, which contributed to the enhancement the tightness and permeability of the seed coat at 8 dpa to physiological maturity (Figure 5B).

### Transcriptional analysis revealed the key genes affecting seed coat permeability

To reveal the mechanism underlying the development stage of seed coat for *E. nutans*, 12 RNA-seq libraries from the early stage of seed coat formation (8 dpa), seed coat maturity (18 dpa), and seed physiological maturity (28 dpa) were constructed. Each development stage had four biological replicates. An overview of the sequencing is presented in Supplementary Table 3. More than 96 and 90% of bases had a *q*-value more than 20 and 30 (an error probability of 0.03%), respectively. The GC-content ranged between 56.35 and 60.53% (Supplementary Table 3). All original FASTQ data files were submitted to the NCBI Sequence Read Archive (SRA) under accession number PRJNA773127. After removing low-quality reads, a total of 570 million clean reads were produced from three samples (Supplementary Table 3). Trinity was used to generate 240,172 transcripts with N50 of 1,418 bp and N90 of 466 bp (Supplementary Table 4). Among them, 128,900 were unigenes, where less than 300 bp has 14,633 unigenes, 300–500 bp has 35,944 unigenes, 500–1,000 bp has 41,569 unigenes, 1,000–2,000 bp has 24,818 unigenes, and >2,000 bp has 11,936 unigenes. A total of 64,988, 83,297, 8,609, 36,154, 48,273, 43,644 and 28,740 unigenes were annotated into the above seven databases, which occupied 50.42, 64.62, 6.68, 28.05, 37.45, 33.86, and 22.3%, respectively (Supplementary Table 5).

**FIGURE 6:**
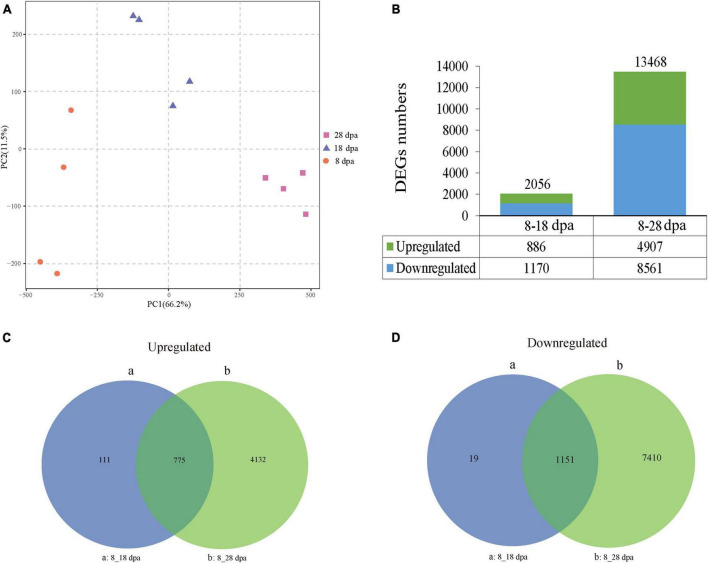
PCA and DEGs during seed development. **(A)** PCA was first used to obtain an overview of RNA-seq of the 8, 18, and 28 dpa conditions. Different plots represent combinations of the first and second principal components (PC1 and PC2). The proportions of explained variance are shown in brackets. **(B)** Numbers of DEGs at 8–28 dpa. The upper and lower parts of each bar represent upregulated and downregulated genes. The total numbers of DEGs are located at the top of the bar in each group. The numbers of up- and downregulated expressed genes are listed at the bottom of the figure. **(C,D)** Venn diagrams show the number of DEGs across three comparisons. (a,b) Show the comparisons of different unigenes identified between 8 dpa vs. 18 dpa and 8 dpa vs. 28 dpa, respectively.

After obtaining the transcriptome data, PCA was performed on 12 samples. PC1 accounted for 66.2%, while PC2 (11.5%) effectively separated the differential samples under the three development stages (Figure 6A). DEGs between the three developmental stages were calculated and normalized using the DESeq method. Adjusted *p*-value < 0.05 was set as the threshold for significant differential expression. We found 2,056 and 13,468 unigenes, which showed differential expression in development samples. Compared with 8 dpa, 886 upregulated unigenes and 1,170 downregulated unigenes were identified at 18 dpa, while 4,907 upregulated unigenes and 8,561 downregulated unigenes were identified at 8 and 28 dpa (Figure 6B). To identify DEGs in different development stages, the overlaps in each comparison were exhibited in a Venn diagram (Figures 6C,D). The overlap of up- and down-regulated genes was between 8 and 18 dpa, which showed 111 and 19 DEGs, suggesting that seed coat development has greatly changed (Figures 6C,D). The overlap of the up- and downregulated unigenes between 8 and 28 dpa were 4,132 and 7,410 DEGs (Figures 6C,D). The results showed that these DEGs were the main factors causing the changes in seed coat permeability.

To identify the primary biological processes that were expressed at different development stages, we showed each GO enrichment analysis of up- and down-regulated DEGs at different physiological stages depending on FDR < 0.05 (Supplementary File 2). On the basis of GO enrichment, we chose the top five most significant accessions to interpret the seed coat permeability changes. At 8 and 18 dpa, the upregulated genes were largely located in the pathway associated with chitin binding and metabolic process while the downregulated genes were mainly located in the pathway associated with cell wall organization (Supplementary Table 6). With seed development, cell wall organization or biogenesis was the significantly enriched pathway that affected testa development. At the same time, xanthine dehydrogenase activity, xanthine catabolic process, and xanthine metabolic process were the three most significantly upregulated pathways during seed coat development (Supplementary Table 6).

**FIGURE 7:**
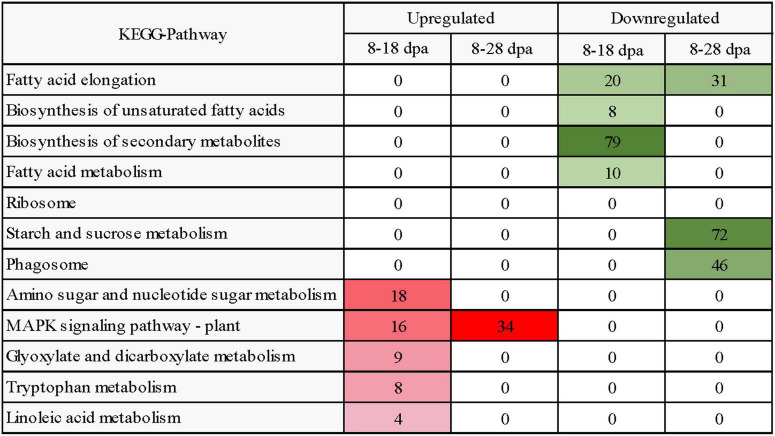
Heatmap diagram reflecting the dynamics of enriched biological process in KEGG analysis during seed development. Enriched processes with FDR < 0.05 were included in the heatmap. Red and green colors represent upregulated and downregulated DEGs, respectively. The intensity of the color reflects the number of DEGs as indicated at each development stage.

KEGG pathway enrichment analysis showed that 396 and 1,160 upregulated DEGs were enriched in 66 and 101 different pathways while comparing 8 with 18 dpa and 8 with 28 dpa (Supplementary File 3). However, 503 and 3,035 downregulated DEGs were involved in 83 and 120 pathways in *E. nutans*, respectively (Supplementary File 3). According to the KEGG pathway analyses, a heatmap reflecting the main enrichment process of seed coat development was constructed (Figure 7). In these pathways, the 8 dpa samples were used as control. Amino sugar and nucleotide sugar metabolism and MAPK signaling pathway—plant pathways had the most upregulated DEGs at 18 dpa, suggesting improved pathway progress in response to seed coat permeability. Meanwhile, the starch and sucrose metabolism, phagosome, and fatty acid elongation pathways had enriched DEGs at 28 days (Figure 7).

Transcription factor (TF) analysis was performed on all identified DEGs, and the genes were randomly selected for quantitative RT-PCR verification (Supplementary Figure 3). The largest group of TFs was the basic helix–loop–helix family, followed by B3, whereas other TFs belonged to the FAR1, NAC, and WRKY families, which including 404, 325, 300, 291, and 284 unigenes (Supplementary Figure 3 and Supplementary File 4). To validate the accuracy of TF sequencing, the relative expression level of six randomly selected unigenes was examined using qRT-PCR analysis (Supplementary Figure 4). These results demonstrated alike expression patterns to the results of RNA-seq.

**FIGURE 8:**
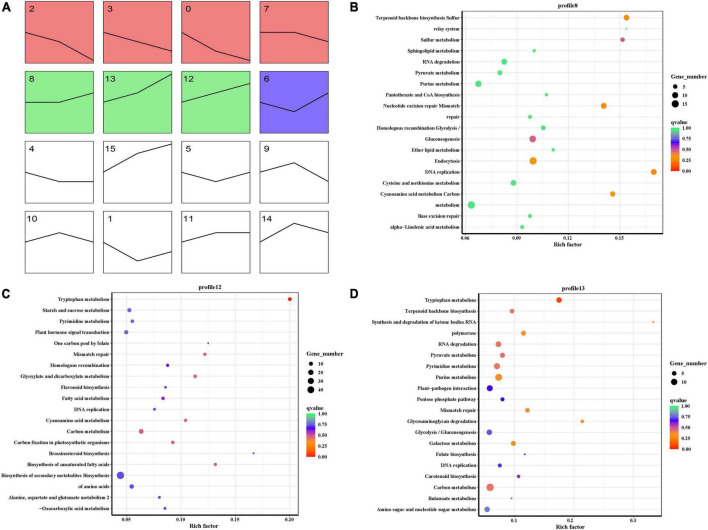
Time series analysis of RNA-seq. **(A)** Overall rendering of gene clustering. **(B)** Profile 8 KEGG pathway enrichment scatterplot. **(C)** Profile 12 KEGG pathway enrichment scatterplot. **(D)** Profile 13 KEGG pathway enrichment scatterplot.

To identify the key gene that contributed to seed permeability, we used time series analysis to explore the RNA-seq results of three developmental stages of *E. nutans*. The analysis produced 16 profiles including 8 profiles that were significantly different (*p* < 0.001). The 8 profiles were separated into 3 groups, and the same color showed the same group. The black lines showed the changing trend of genes with seed developments (Figure 8A). At 8, 18, and 28 dpa, we focused on the gene showing an increasing trend. The results showed that profile 8, 12, and 13 had the same trends, and all the clustered genes were analyzed by KEGG (Figures 8B–D). Tryptophan metabolism was the most significantly enriched pathway, which is similar to that of heatmap analysis.

### Correlation between differentially expressed metabolites and differentially expressed genes and their insights into seed coat development in *Elymus nutans*

To explore the regulatory genes affecting metabolites, we conducted the analysis into two parts. One is to use the significant pathway enriched by metabolites to find the same pathway with RNA-seq and then identify the DEGs in this pathway. The other is to analyze the correlation between all DEMs identified by MS2 and DEGs obtained by RNA-seq analysis. The relative quantitation and FPKM values of the two omics were used to find the genes.

**FIGURE 9:**
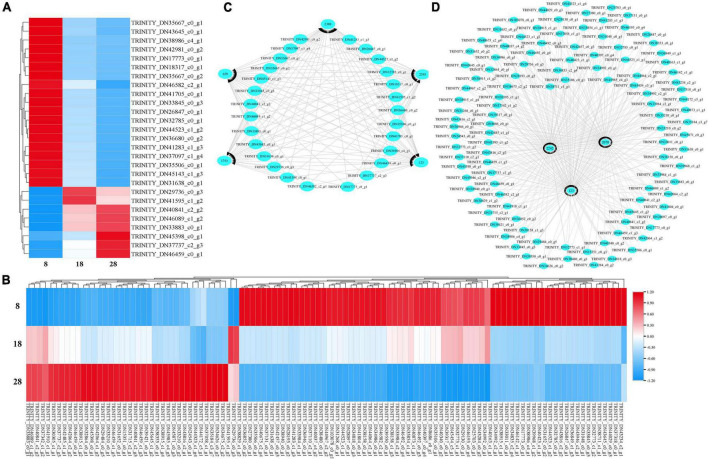
Heatmap and cytoscape shows the DEGs in KEGG pathways and the interaction between the key metabolites and their associated DEGs that were significantly enriched under metabolic analysis. **(A,B)** All DEGs identified at 8 vs. 18 dpa and 8 vs. 28 dpa, respectively. **(C,D)** Key genes between 8 and 18 dpa, respectively. 1,753: thiamine, 2,308: alpha-lactose, 123: salicylic acid (SA), 2,242: docosapentaenoic acid, 2,070: eicosapentaenoic acid, 659: L-glutamic acid, 2,395: DL-alpha-tocopherol.

For the first analysis, 27 genes were annotated into ABC transporters, phenylalanine metabolism, ubiquinone and other terpenoid-quinone biosynthesis, and plant hormone signal transduction at 8 with 18 dpa (Figure 9A). When we compared the immature seeds with mature seeds, 110 DEGs were annotated in the biosynthesis of UFA, sulfur relay system, phenylalanine metabolism, and plant hormone signal transduction (Figure 9B). To explore the regulatory mechanism of the formation of key compounds affecting seed coat permeability, pearson correlation coefficient was used to analyze the correlation between metabolites and genes among samples. We built a metabolite-gene correlation network for two comparisons using 76 and 78 DEMs under the POS and 67 and 80 DEMs under the NEG and 2,056 and 13,468 DEGs in each comparison (8 and 18 dpa and 8 and 28 dpa), respectively. The DEMs in key pathways and the corresponding DEGs were analyzed and visualized by Cytoscape. The results show that depending on different combination, 659 (L-glutamic acid), 1753 (thiamine), 2308 (alpha-lactose), 2395 (DL-alpha-tocopherol) and 123 (SA) had the relationship with those genes which included in 27 DEGs (Figure 9C). However, 2070 (eicosapentaenoic acid), 2242 (docosapentaenoic acid), and 123 (SA) had the relationship with 110 DEGs (Figure 9D).

### Response of key unigenes to thiamine and salicylic acid formation

On the basis of the correlation coefficient between metabolites and genes, we chose *p*-value less than 0.05 and correlation coefficient —r— > 0.8. Thiamine and SA had the corresponding genes that met the screening criteria. In the comparison combination of 8 and 18 dpa, 14 and 4 genes had a correlation coefficient —r— > 0.8, which were related to thiamine and SA, respectively (Supplementary File 5). For the immature and mature seed coats (8 and 28 dpa), 14 genes contributed to seed coat permeability development when SA were significantly deposited in the seed coat (Supplementary File 5).

**FIGURE 10:**
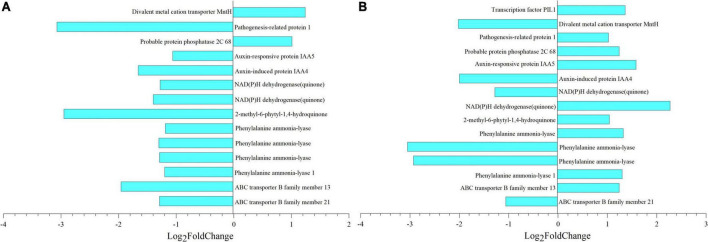
Metabolic and gene expression changes associated with seed coat development of *E. nutans*. Column diagram representing the accumulation of key metabolites that were significantly associated with DEGs. **(A,B)** Differential multiples of key genes between 8 vs. 18 dpa and 8 vs. 28 dpa, respectively. 1,753, thiamine; 123, salicylic acid (SA).

After deduplication of all DEGs obtained from the analysis, the fold change of each gene in different comparisons was analyzed, and selected genes with —Log_2_ Fold Change— greater than 1 were mapped (Figure 10). Taking 8 dpa as control, 14 and 15 genes regulated thiamine and SA, respectively. Meanwhile, pathogenesis-related protein 1 (*PR1*) which regulated thiamine formation had Log_2_ Fold Change of –3.07 (Figure 10A). In the meantime, phenylalanine ammonia-lyase (*PAL*) which regulated SA formation had the —Log_2_ Fold Change— greater than 3 especially when compared 8 with 28 dpa (Figure 10B).

## Discussion

### Changes in seed coat permeability during seed development

With seed development, the seed viability and 1,000-seed weight significantly increased, while the moisture content, conductivity, and imbibition rate showed the opposite results. In particular, EC did not change at a certain stage of seed development (Figure 1). This result is similar to that of some gramineous forages such as *E. sibiricus*, *Roegneria nutans*, *Festuca sinensis*, *Achnatherum inebrians*, and *Hordeum vulgare* var. *nudum* (Zhou et al., 2013a). The seed coat is believed to contain a semi-permeable layer, which leads to decreased seed permeability. According to anatomical microscopic observation, this structure is formed by the thickening of the peripheral cell wall of lateral cells of the inner integument (Zhou et al., 2013b). In our study, this layer prevents the infiltration of large molecules, including lanthanum nitrate and fluorescent dyes (Figure 2 and Supplementary Figure 2).

The semi-permeable layer is a common structure in grass seeds, which affects seed vigor detection using the EC method (Lv et al., 2017). This structure is located in the seed coat. Considering that the semi-permeable layer is a very thin layer and is difficult to separate from the testa, it can’t be extracted for analysis. Therefore, we regarded the barrier function of the semi-permeable layer to be a function of the seed coat and considered that the formation of this layer is the main reason for the rapid decline of seed coat permeability. However, previous studies have demonstrated that the semi-permeable layer is a lifeless tissue, and its function is due to the deposition of chemical substances on the structure (Zhou et al., 2013b). Histochemical staining showed that the substances causing the change in seed coat permeability included lipid, pectin, and cellulose (Zhou et al., 2013a). However, detailed reports on the chemical composition of the seed coat are lacking. Therefore, this study focused on the specific components that caused changes in seed coat permeability through high-throughput metabolomic analysis. The results showed that the main substances causing the decrease of seed coat permeability are thiamine and SA (Figure 5).

### Differential metabolites causing changes in seed coat permeability

Through metabolomics analysis, a total of 1,887 metabolites were identified, including 4-coumaric acid, L-glutamic acid, DL-alpha-tocopherol, thiamine, alpha-lactose, and SA which were the main differentially abundant compounds with seed development. In *Styrax tonkinensis* seeds developing, a total of 187 and 1,556 metabolites were obtained and carboxylic acids and derivatives, flavonoids, fatty acid were the main components (Wu et al., 2020). However, we found that the significant increase of thiamine and SA during early seed development was the main reason for the decrease of seed coat permeability (Figure 5A). As one of the B-complex vitamins, thiamine is indispensable to provide energy for organs with active cell division and plays predominantly antioxidant roles in cellular physiological metabolic processes under environmental stresses (Asensi-Fabado and Munné-Bosch, 2010; Rosado-Souza et al., 2020). Thiamine biosynthesis is a highly regulated process involving light, stress, circadian rhythms, and the cofactor pyrophosphate (Khozaei et al., 2015). It is known to enhance plant defense by priming, but its effect on seed development is rarely reported (Nagae et al., 2016; Wang et al., 2016). In *Lathyrus sativus*, *Lotus japonicus*, and *Phyllanthus amarus*, the thiamine content will increase with seed development, which is similar to the results in this experiment (Lisiewska et al., 2003; Okiki et al., 2015; Nagae et al., 2016). In the whole process of seed growth and development, a good defense system is the key to ensure the healthy growth of seeds. As a physical barrier, the seed coat plays a pioneering role in protection. The presence of thiamine indicates that the seed coat is capable of resisting the external environment during seed growth.

In addition to thiamine, SA is also the main chemicals deposited in the seed coat during the early stages of seed development. SA is a kind of small molecular phenols widely distributed in higher plants and is also an important plant hormone, which can participate in the response to stress through complex signal transduction network (Ma et al., 2017). SA is known to increase in many patho systems upon infection with viruses, fungi, insects, and bacteria (Lefevere et al., 2020). In our research, the relative quantitative value of SA showed a significant upward trend among different comparisons (Figure 5). This result reflects that the seed coat accumulates endogenous hormones during the development process to enhance its role in protecting the seed embryo from external environmental interference, so as to ensure the healthy development of the seed.

### Key genes regulating seed coat permeability

According to the transcriptome analysis, 2056 and 13,468 unigenes were observed, which showed differential expression in development samples (Figure 6B). Through joint analysis of key genes identified by transcription and key substances identified by metabolism, we found the key genes that regulated the changes in seed coat permeability, including *PR1* and *PAL* (Figure 10 and Supplementary File 5). PRs are a great protein superfamily in plants and play important roles in plant response to various biotic and abiotic stresses (Tang et al., 2019; Ghorbel et al., 2021). In tobacco, thiamine homeostasis in plants was regulated by the transketolase protein which provided the precursor for synthesis of intermediates and to enable plants to produce thiamine and thiamine pyrophosphate for growth and development (Khozaei et al., 2015). In our research, *PR1* has the highest —log_2_Fold Change— multiple of 3.07, and its correlation coefficient with thiamine was –0.88 (Figure 10A and Supplementary File 5). The results showed that the gene was closely related to the substance that caused the change of seed coat permeability, so it might be a key gene in regulating the change of seed coat permeability.

At the same time, *PAL* is one of the genes with the greatest difference in seed coat development. It occurs mainly in mature seed coat and regulates the formation of SA (Figure 10B and Supplementary File 5). It is widely accepted that plants possess PAL pathway to synthesize SA because PAL is an upstream enzyme that leads to many other possibly defense-related compounds (Lefevere et al., 2020). *PAL* genes are differentially expressed between plant tissues and induction upon infection with a distinct pathogen (Tonnessen et al., 2015). Seed coat development plays an important role in the whole plant growth process, which makes seeds have strong adaptability to adverse natural environment. The reason may be the existence of a large number of defense proteins regulating the course of disease, protecting the development of seeds, and this result is similar to the previous studies.

In this study, the formation mechanism of seed coat permeability was elucidated using metabolome and transcriptome data in *E. nutans*. Two key metabolites including thiamine and SA were found to affect seed coat permeability. Meanwhile, *PR1* and *PAL* play an important role in regulating the formation of compounds. This study provides not only insights into the changes in seed coat permeability caused by compounds deposited on the seed coat, but also candidate gene resources for seed coat development. The results will supply a great significance value to seed production and quality evaluation.

## Data availability statement

The RNA-Seq data and the datasets presented in this study can be found at the NCBI repository, accession number PRJNA773127, https://www.ncbi.nlm.nih.gov/bioproject/PRJNA773127.

## Author contributions

JZ conceived, designed and performed the experiments, and wrote the manuscript. YL, XW, and YJL took the samples and performed parts of the experiments. RD-S revised and edited the manuscript. MW checked the experiments data. SQ made substantial contributions to the data analysis. ZG provided some suggestions. FY conceived the experiments and modified the manuscript. All authors have read and approved the final manuscript.

## Footnotes

1
http://www.kegg.jp

2
http://www.hmdb.ca/

3
http://metlin.scripps.edu/index.php

4
http://www.ontobee.org/ontology/CHEBI

5
https://pubchem.ncbi.nlm.nih.gov/

6
https://github.com/COMBINE-lab/salmon

7
http://www.bioinformatics.org/cd-hit/

8
http://planttfdb.gao-lab.org/

## Supplementary material

Supplementary Figure 1 Anatomical microsturcture of seed coating at different developmental stages of E. nutans. **(A)** The section of the ovary at 8 dpa with nucellus cell (nu), two layers of the inner integument (ii), and the pericarp (pe). **(B)** The seed coat (sc) had formed at 18 dpa. The nucellus cell differentiated into aleurone (al) and endosperm (en) cells. **(C)** In addition to the embryo, mature seeds include pericarp, seed coat, aleurone layer, and endosperm cells at 28 dpa.

Supplementary Figure 2 SEM image of seeds and EDX detection of different parts of seeds. **(A)** Scanning electron microscopy image of seed structure. **(B–F)** Distribution of lanthanum deposition in different positions. Oval boxes represent the presence of lanthanum.

Supplementary Figure 3 TFs that were differentially expressed under different development stages in Elymus nutans.

Supplementary Figure 4 qRT-PCR analysis of the relative expression level of six unigenes.
