# Supplementary material for: Analysis of Elymus nutans seed coat development elucidates the genetic basis of metabolome and transcriptome underlying seed coat permeability characteristics
Source: Front Plant Sci. 2022 Aug 18;13:970957. doi: 10.3389/fpls.2022.970957 (PMC9437961; doi:10.3389/fpls.2022.970957)
Supplement: Supplementary file 6 [file Table_1.DOCX]

**Supplementary Table S1.** The primers of six unignes and *EnACT*

| Gene | Forward primer | Reverse primer |
| --- | --- | --- |
| TRINITY_DN29686_c1_g1 | GGCGTTGTTGAGGTTGAGAT | CCAGGTTGACAGCTTGATGAA |
| TRINITY_DN42192_c1_g2 | CAAACGCAAGTTGATACAGGACG | GTGTCTCCACCCAGGAACCC |
| TRINITY_DN42591_c1_g1 | TTCCGAAGAGGCAGCAGATAT | CACTGGCGTAGAAGCAGATTG |
| TRINITY_DN35932_c1_g1 | CGATGATGACGATGACGATGATG | CAGAATGGCACGAGGAAGATG |
| TRINITY_DN37560_c0_g1 | AGATAGATGGTGTGCCTCTGT | GCCAGTAAGTAGCAGCAAGATAA |
| TRINITY_DN29576_c3_g2 | CAAGCAGAGCAGAGTGACAAACCA | GTCATGGGACGCCGAAACG |
| *EnACTIN* JF683844 | GTCCTCTTCCAGCCATCCAT | GTTTCCATACAAGTCCTTCCTGAT |
