# Supplementary material for: Analysis of Elymus nutans seed coat development elucidates the genetic basis of metabolome and transcriptome underlying seed coat permeability characteristics
Source: Front Plant Sci. 2022 Aug 18;13:970957. doi: 10.3389/fpls.2022.970957 (PMC9437961; doi:10.3389/fpls.2022.970957)
Supplement: Supplementary file 7 [file Table_2.DOCX]

**Supplementary Table S2.** The metabolites of KEGG significantly enriched upregulated pathway

| Ion mode | Metabolites ID | 8-18 dpa | |  | 8-28 dpa | |  |
| --- | --- | --- | --- | --- | --- | --- | --- |
|  |  | VIP | *P*-value | Fold change | VIP | *P*-value | Fold change |
| POS | 2308 | 1.3982 | 0.0001 | 1.5378 | 1.2875 | 0.0001 | 2.1003 |
|  | 659 | 1.3655 | 0.0009 | 1.5483 | -- | -- | -- |
|  | 262 | 1.1173 | 0.0234 | 1.4135 | -- | -- | -- |
|  | 1753 | 1.3685 | 0.0005 | 1.8836 | 1.2300 | 0.0012 | 2.317 |
|  | 1773 | 1.1303 | 0.0207 | 1.3174 | -- | -- | -- |
|  | 2242 | 1.3140 | 0.0021 | 1.3427 | 1.2713 | 0.0002 | 1.4911 |
|  | 2070 | 1.3260 | 0.0017 | 1.3809 | 1.2439 | 0.0007 | 1.5481 |
| NEG | 183 | 1.5306 | 0.0004 | 1.6577 | 1.2596 | 0.0034 | 2.9529 |
|  | 123 | 1.4682 | 0.0014 | 1.4598 | 1.2100 | 0.0073 | 1.926 |
|  | 241 | 1.3380 | 0.0127 | 1.4623 | -- | -- | -- |
|  | 2395 | 1.3963 | 0.0082 | 1.915 | -- | -- | -- |
