# Supplementary material for: Analysis of Elymus nutans seed coat development elucidates the genetic basis of metabolome and transcriptome underlying seed coat permeability characteristics
Source: Front Plant Sci. 2022 Aug 18;13:970957. doi: 10.3389/fpls.2022.970957 (PMC9437961; doi:10.3389/fpls.2022.970957)
Supplement: Supplementary file 8 [file Table_3.DOCX]

**Supplementary Table S3.** Overview of the sequencing

| Gategory | 8 dpa | | | | 18 dpa | | | | 28 dpa | | | |
| --- | --- | --- | --- | --- | --- | --- | --- | --- | --- | --- | --- | --- |
|  | 1-1 | 1-2 | 1-3 | 1-4 | 2-1 | 2-2 | 2-3 | 2-4 | 3-1 | 3-2 | 3-3 | 3-4 |
| Raw reads | 51185644 | 46507326 | 47130376 | 53514362 | 50712822 | 44838218 | 45596220 | 54575676 | 50540626 | 44970424 | 50217192 | 45097074 |
| Clean reads | 49501188 | 45156994 | 45775324 | 52154406 | 49168460 | 43599114 | 44570694 | 53174808 | 49645756 | 43988080 | 49213018 | 44214304 |
| Cleanbases(Gb) | 7.43 | 6.77 | 6.87 | 7.82 | 7.38 | 6.54 | 6.69 | 7.98 | 7.45 | 6.6 | 7.38 | 6.63 |
| Q20 (%) | 96.03% | 96.97% | 96.92% | 96.89% | 97.18% | 96.87% | 96.99% | 96.92% | 97.22% | 97.30% | 97.34% | 97.34% |
| Q30 (%) | 90.60% | 92.54% | 92.50% | 92.37% | 92.99% | 92.41% | 92.57% | 92.49% | 92.95% | 93.19% | 93.27% | 93.28% |
| GC (%) | 57.30% | 56.35% | 57.61% | 57.14% | 59.32% | 58.85% | 59.38% | 58.84% | 59.96% | 59.50% | 59.71% | 60.53% |
| Error (%) | 0.03% | 0.03% | 0.03% | 0.03% | 0.03% | 0.03% | 0.03% | 0.03% | 0.03% | 0.03% | 0.03% | 0.03% |
