# Supplementary material for: Analysis of Elymus nutans seed coat development elucidates the genetic basis of metabolome and transcriptome underlying seed coat permeability characteristics
Source: Front Plant Sci. 2022 Aug 18;13:970957. doi: 10.3389/fpls.2022.970957 (PMC9437961; doi:10.3389/fpls.2022.970957)
Supplement: Supplementary file 9 [file Table_4.DOCX]

**Supplementary Table S4.** Summary statistics of *Elymus nutans* transcriptome assemblies

| Nucleotides Length (bp) | Transcripts | Unigenes |
| --- | --- | --- |
| ＜300 | 24791 | 14633 |
| 300-500 | 54863 | 35944 |
| 500-1000 | 75882 | 41569 |
| 1000-2000 | 57801 | 24818 |
| ＞2000 | 26835 | 11936 |
| Total | 240172 | 128900 |
| Shortest length | 201 bp | 201 bp |
| Median length | 711 bp | 607 bp |
| Longest length | 15926 bp | 15926 bp |
| N50 | 1418 bp | 1273 bp |
| N90 | 466 bp | 417 bp |
