# Supplementary material for: Analysis of Elymus nutans seed coat development elucidates the genetic basis of metabolome and transcriptome underlying seed coat permeability characteristics
Source: Front Plant Sci. 2022 Aug 18;13:970957. doi: 10.3389/fpls.2022.970957 (PMC9437961; doi:10.3389/fpls.2022.970957)
Supplement: Supplementary file 10 [file Table_5.DOCX]

**Supplementary Table S5.** Unigene information annotated in different databases

| Public Database | Number of Unigenes | Percentage (%) |
| --- | --- | --- |
| Annotated in Nr | 64988 | 50.42 |
| Annotated in Nt | 83297 | 64.62 |
| Annotated in KEGG | 8609 | 6.68 |
| Annotated in Swiss Prot | 36154 | 28.05 |
| Annotated in Pfam | 48273 | 37.45 |
| Annotated in GO | 43644 | 33.86 |
| Annotated in KOG | 28740 | 22.3 |
| Annotated in all Databases | 5170 | 4.01 |
| Annotated in at least one Database | 95236 | 73.88 |
| Total Unigenes | 128900 | 100 |
