# Supplementary material for: Analysis of Elymus nutans seed coat development elucidates the genetic basis of metabolome and transcriptome underlying seed coat permeability characteristics
Source: Front Plant Sci. 2022 Aug 18;13:970957. doi: 10.3389/fpls.2022.970957 (PMC9437961; doi:10.3389/fpls.2022.970957)
Supplement: Supplementary file 11 [file Table_6.DOCX]

**Supplementary Table S6.** DEGs significantly enriched in 5 up- and downregulated pathways of GO

|  | downregulated |  |  |  |  | upregulated |  |  |  |
| --- | --- | --- | --- | --- | --- | --- | --- | --- | --- |
|  | GO_accession | Description | Corrected_pValue | DEG_item |  | GO_accession | Description | Corrected_p  Value | DEG_item |
| 8-18  dpa | GO:0005576 | extracellular region | 2.49E-17 | 65 |  | GO:0045735 | nutrient reservoir activity | 8.3729E-20 | 24 |
|  | GO:0071554 | cell wall organization or biogenesis | 9.23E-13 | 52 |  | GO:0008061 | chitin binding | 6.3703E-15 | 17 |
|  | GO:0005618 | cell wall | 9.40E-12 | 42 |  | GO:0006026 | aminoglycan catabolic process | 6.3888E-15 | 17 |
|  | GO:0030312 | external encapsulating structure | 1.50E-11 | 42 |  | GO:0006030 | chitin metabolic process | 6.3888E-15 | 17 |
|  | GO:0016747 | transferase activity, transferring acyl groups other than amino-acyl groups | 3.43E-11 | 48 |  | GO:0006032 | chitin catabolic process | 6.3888E-15 | 17 |
| 8-28  dpa | GO:0005576 | extracellular region | 1.3075E-45 | 280 |  | GO:0045735 | nutrient reservoir activity | 2.778E-10 | 27 |
|  | GO:0071554 | cell wall organization or biogenesis | 2.6052E-35 | 228 |  | GO:0004854 | xanthine dehydrogenase activity | 4.7754E-10 | 17 |
|  | GO:0030312 | external encapsulating structure | 4.3229E-32 | 181 |  | GO:0009115 | xanthine catabolic process | 4.7754E-10 | 17 |
|  | GO:0005618 | cell wall | 1.9646E-31 | 177 |  | GO:0046110 | xanthine metabolic process | 4.7754E-10 | 17 |
|  | GO:0009534 | chloroplast thylakoid | 7.2856E-29 | 139 |  | GO:0005506 | iron ion binding | 7.4924E-10 | 91 |
