# Supplementary figures and images for: Analysis of Elymus nutans seed coat development elucidates the genetic basis of metabolome and transcriptome underlying seed coat permeability characteristics
Source: Front Plant Sci. 2022 Aug 18;13:970957. doi: 10.3389/fpls.2022.970957 (PMC9437961; doi:10.3389/fpls.2022.970957)

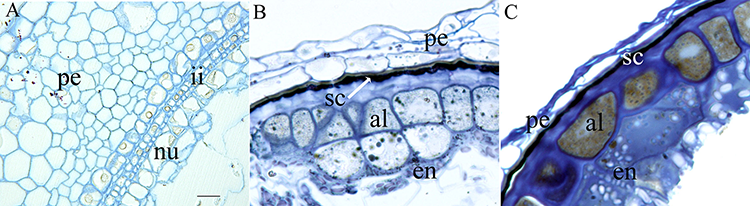

Supplement: Supplementary Figure 1 — Anatomical microsturcture of seed coating at different developmental stages of E. nutans. (A) The section of the ovary at 8 dpa with nucellus cell (nu), two layers of the inner integument (ii), and the pericarp (pe). (B) The seed coat (sc) had formed at 18 dpa. The nucellus cell differentiated into aleurone (al) and endosperm (en) cells. (C) In addition to the embryo, mature seeds include pericarp, seed coat, aleurone layer, and endosperm cells at 28 dpa. [file Image_1.TIF]

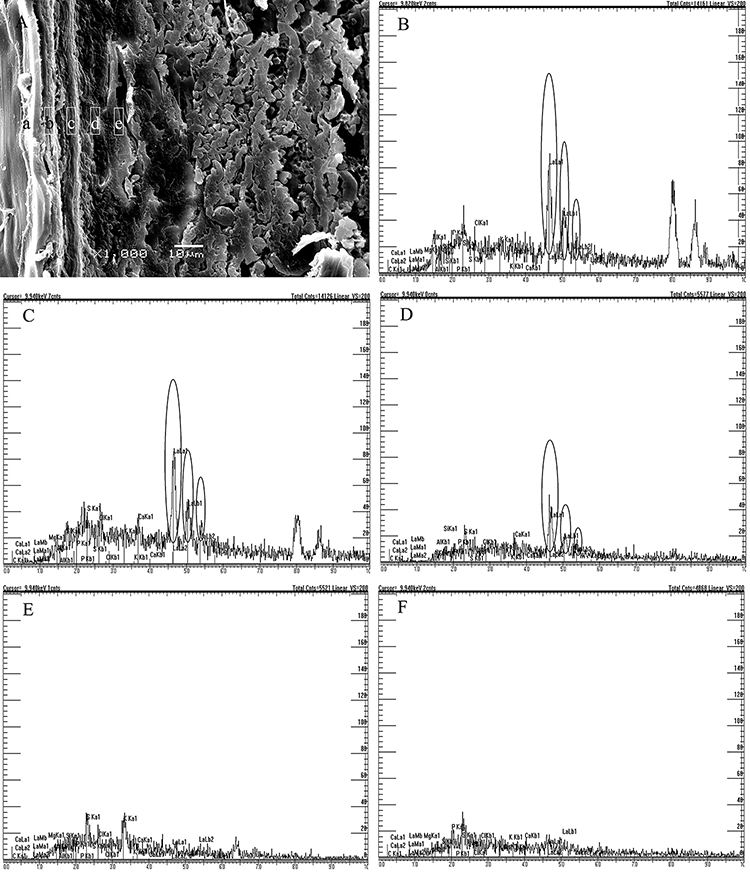

Supplement: Supplementary Figure 2 — SEM image of seeds and EDX detection of different parts of seeds. (A) Scanning electron microscopy image of seed structure. (B–F) Distribution of lanthanum deposition in different positions. Oval boxes represent the presence of lanthanum. [file Image_2.TIF]

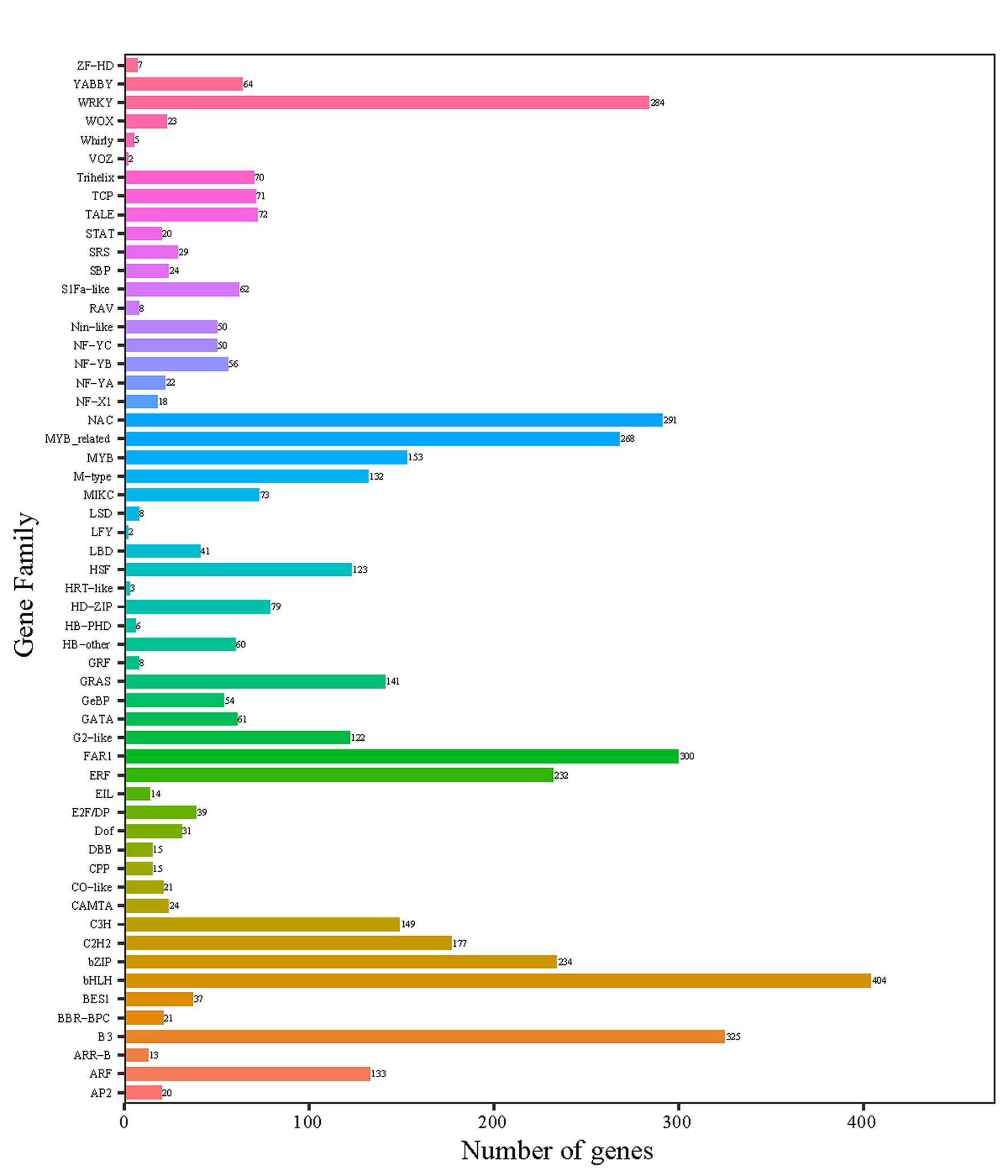

Supplement: Supplementary Figure 3 — TFs that were differentially expressed under different development stages in Elymus nutans. [file Image_3.TIF]

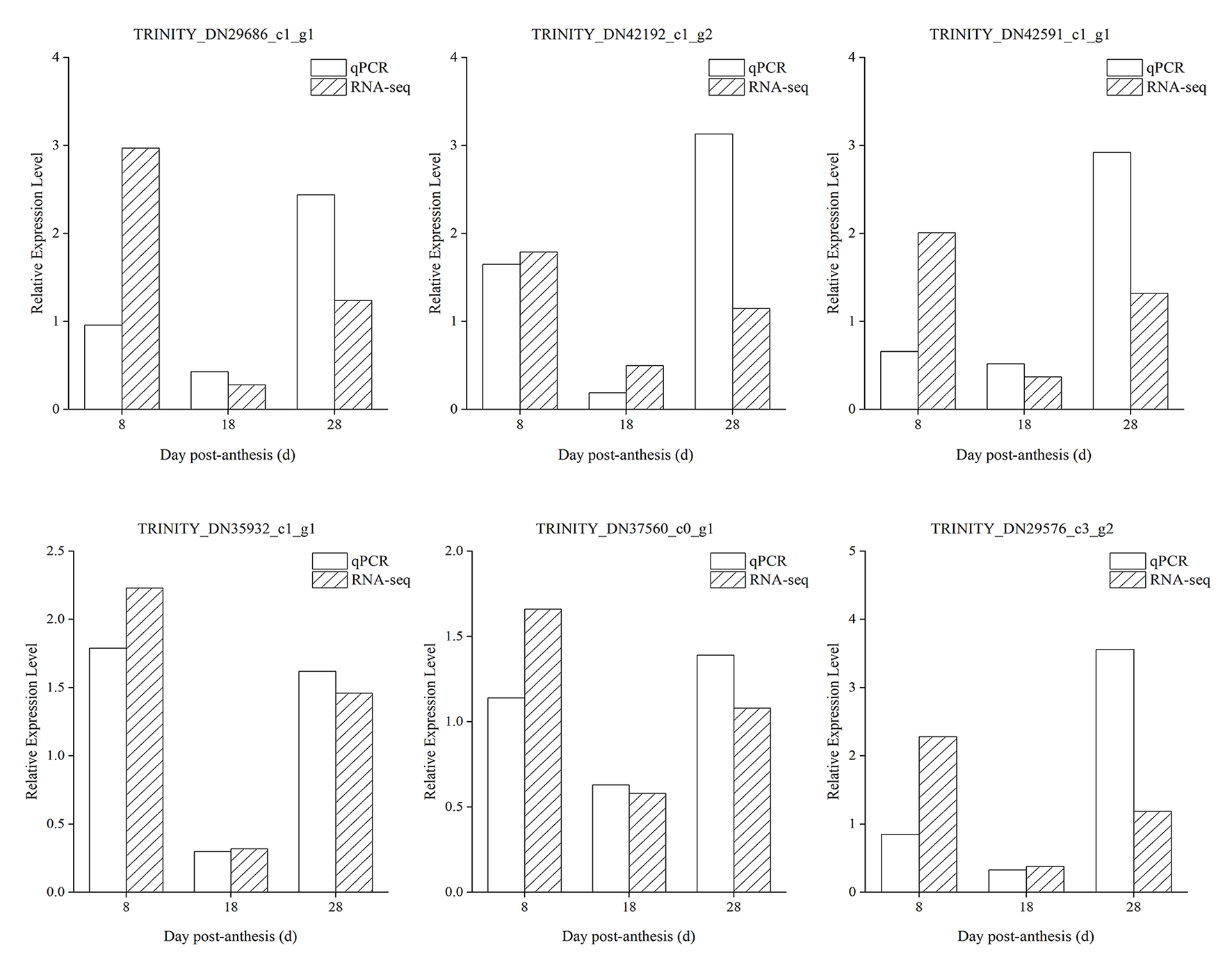

Supplement: Supplementary Figure 4 — qRT-PCR analysis of the relative expression level of six unigenes. [file Image_4.TIF]
